# Supplementary material for: Delving deeper: Relating the behaviour of a metabolic system to the properties of its components using symbolic metabolic control analysis
Source: PLoS One. 2018 Nov 28;13(11):e0207983. doi: 10.1371/journal.pone.0207983 (PMC6261606; doi:10.1371/journal.pone.0207983)
Supplement: S1 Data Files — (ZIP) [file pone.0207983.s001.zip › instructions.pdf]

Delving deeper: Relating the behaviour of a metabolic system to the properties of its components using symbolic metabolic control analysis (Supplementary data)

Carl D. Christensen, Jan-Hendrik S. Hofmeyr, and Johann M. Rohwer

October 10, 2018

Contents

1 Introduction **2**

2 Summary of included files **2**

3 Installing PySCeSToolbox **2**

3.1 Windows . . . 3

3.2 Mac OS X (MacOS) . . . 3

3.3 Linux . . . 4

4 Running PySCeSToolbox **4**

4.1 Running the example notebook . . . 5

1 Introduction

This document includes instructions for installing and running PySCeSToolbox with the goal of running the Jupyter notebook included as part of "S1 Example Analysis" that serves to illustrate how to perform symbolic control analysis and control pattern analysis as demonstrated in the main text of "Delving deeper: Relating the behaviour of a metabolic system to the properties of its components using symbolic metabolic control analysis". Further instructions are given within the notebook itself.

2 Summary of included files

A number of files are included as supplementary material that serve to illustrate how the analysis presented in the main text was performed. These files include a Jupyter notebook file and supporting model and data files. A description of each of these files and their purpose is provided in Table 1 below.

Table 1: Included files

`hoefnagel_ratio_FRN.psc`	The PySCeS model of pyruvate branch metabolism with NADH/NAD$^+$ as a parameter that used to produce the results presented in the main text. This file is required by `example_notebook.py`.
`hoefnagel_ratio.psc`	The PySCeS model of pyruvate branch metabolism with NADH/NAD$^+$ as a free variable that used to produce the results presented in the main text. This file is not required by `example_notebook.py`.
`hoefnagel_ratio.reqn`	A file that defines how reactions of the pyruvate branch metabolism system should be split into their binding and mass action components. This file is required by `example_notebook.py`.
`example_notebook.ipynb`	A notebook that replicates a selection of results found in the main text.
`symca_data.pkl`	Pre-generated control coefficient expression data for analysis of `hoefnagel_ratio_FRN.psc` using `SymCA`. This file is required by `example_notebook.py`.
`graph_layout.dict`	The layout file for the model scheme of `hoefnagel_ratio_FRN.psc` and `hoefnagel_ratio.psc`. This file is required by `example_notebook.py`.
`additional_functions.py`	Additional code for running `example_notebook.py`.

3 Installing PySCeSToolbox

PySCeSToolbox is compatible with Mac OS X, Linux, and Windows. Operating system-specific instructions are discussed in the sections below. We have made special effort to provide as detailed instructions as possible, assuming a clean installation of each operating system prior to installation of PySCeSToolbox, and relatively limited knowledge of Python. If further assistance is required, please contact the authors.

Abbreviated requirements: PySCeSToolbox has a number of requirements that must be met before installation can take place. Fortunately most requirements, save for a few exceptions (as

discussed in the operating system-specific sections), will be taken care of automatically during installation. An abbreviated list of requirements follows:

- A Python 2.7 installation
- The full SciPy Stack (see http://scipy.org/install.html)
- PySCeS (see http://pysces.sourceforge.net)
- Maxima (see http://maxima.sourceforge.net)
- Jupyter Notebook (version in the 4.x.x series or greater)

3.1 Windows

Windows requires the manual installation of **Python 2.7** and **Maxima**. Installation was tested on Windows 8 and 10.

Python: For Windows users (especially those unfamiliar with Python) we recommend using the Anaconda Python distribution (https://www.continuum.io/downloads#windows). This is a low fuss solution that will install Python on you system *together with many of the packages necessary for running PySCeSToolbox*. Download the appropriate **Python 2.7** package from the download page (most probably the 64bit edition) and follow the instructions of the installation wizard.

Maxima: Maxima is necessary for generating control coefficient expressions using SymCA. For the sake of convenience, we have included pre-generated expressions for use in the included Jupyter notebook
`example_notebook.ipynb`. Thus, this component is only necessary if you would like to generate new control coefficient expressions or to run the included `benchmark_script.py`.

The latest version of Maxima can be downloaded and installed from the Windows download page at http://maxima.sourceforge.net/download.html.

Windows might also require the path to `maxima.bat` to be defined in the `psctb_config.ini` file (found at `C:\Pysces\psctb_config.ini` by default). The default path included in `psctb_config.ini` is set as `C:\Program Files?\Maxima?\bin\maxima.bat`, where the question marks are wildcards (since the specific path will depend on the version of Maxima). If Maxima has been installed to a user specified directory, the correct path to the `maxima.bat` file must be specified here.

PySCeSToolbox: Now you are ready to install PySCeSToolbox. In the Anaconda Command Prompt (Start → Anaconda Command Prompt), simply type in and execute the command:

```
pip install pyscestoolbox
```

As previously mentioned, this will automatically download both PySCeSToolbox and any outstanding requirements.

3.2 Mac OS X (MacOS)

Mac OS X requires the manual installation of **Maxima**. While OS X comes pre-installed with **Python 2.7** we still recommend installing a Python distribution such as Anaconda as it will take care of many of the SciPy stack requirements. Installation was tested on MacOS Sierra.

Python: One of the most simple ways to get Python on your system is to install the Anaconda Python distribution (https://www.continuum.io/downloads#macos). Download either of the Python 2.7 installers from the download page and follow the provided instructions.

Maxima: Maxima is necessary for generating control coefficient expressions using SymCA. For the sake of convenience, we have included pre-generated expressions for use in the included Jupyter notebook
`example_notebook.ipynb`. Thus, this component is only necessary if you would like to generate new control coefficient expressions or to run the included `benchmark_script.py`.

The latest version of Maxima can be downloaded and installed from the MacOS download page at http://maxima.sourceforge.net/download.html. We recommend the VTK version of Maxima. After downloading and installing the Maxima dmg, the following lines must be added to your

.bash_profile file (note that the second line must not be broken in your .bash_profile file as it is below):

```
export M_PREFIX=/Applications/Maxima.app/Contents/Resources/opt
export PYTHONPATH=$M_PREFIX/Library/Frameworks/Python.framework/Versions/2.7/lib/
  python2.7/site-packages/:$PYTHONPATH
export MANPATH=$M_PREFIX/share/man:$MANPATH
export PATH=$M_PREFIX/bin:$PATH
alias maxima=rmaxima
```

PySCeSToolbox: Now you are ready to install PySCeSToolbox. In the Terminal (or iTerm) simply type in and execute the command:

```
pip install pyscestoolbox
```

As previously mentioned, this will automatically download both PySCeSToolbox and any outstanding requirements.[1]

3.3 Linux

Linux only requires the manual installation **Maxima**. Most Linux systems come pre-installed with **Python 2.7**, however a Python distribution such as Anaconda may be used instead. Installation was tested on Ubuntu 16.04.

Python: We assume that your system comes with Python 2.7 and with pip (necessary for installing Python packages that are not available in your OS's repositories). Pip may be installed from your OS's repositories or by following the instructions found at https://pip.pypa.io/en/stable/installing/.

Maxima: Maxima is necessary for generating control coefficient expressions using SymCA. For the sake of convenience, we have included pre-generated expressions for use in the included Jupyter notebook
example_notebook.ipynb. Thus, this component is only necessary if you would like to generate new control coefficient expressions or to run the included benchmark_script.py. Maxima can be installed from your repositories, if available, otherwise the latest packages can be downloaded from the Linux link at http://maxima.sourceforge.net/download.html.

PySCeSToolbox: Now you are ready to install PySCeSToolbox. In the terminal simply type in and execute the command:

```
pip install pyscestoolbox
```

4 Running PySCeSToolbox

Once the requirements are fulfilled, the instructions for running PySCeSToolbox are operating system agnostic. However, for the remainder of this section we will refer to the terminal (Linux and OS X) and the Anaconda command prompt (Windows) as the terminal.

Note that the notebook below was coded in such a way that all the required files and the scripts themselves must be located in the same directory from which the notebook is executed. In the case where the location of a model for a specific script is not explicitly defined as they are in the supplied scripts, then PySCeS will load models from the C:\Pysces\psc directory on Windows or the ~/Pysces/psc/ directory in Linux and OS X.

[1] You may encounter an error during the installation of PySCeSToolbox relating to the removal of temporary files on OS X or MacOS. This does not impact on the functioning of PySCeSToolbox at all, and we mean to address this bug in the future.

4.1 Running the example notebook

If you have never started a Jupyter Notebook on your system, or have never used Jupyter's widgets system, the following command must be executed to enable the widget extension prior to starting a Jupyter Notebook session (it only has to be executed once):

```
jupyter nbextension enable --py --sys-prefix widgetsnbextension
```

Thereafter a Jupyter Notebook session can be started from within the directory that contains the `example_notebook.ipynb` file and the other files defined in Table 1 using the command:

```
jupyter notebook
```

This will open up the default browser with a notebook session showing the contents of the current directory. Selecting `example_notebook.ipynb` from this window will open it in a new browser tab. Further instructions are contained within the notebook itself. Additional information about PySCeSToolbox's functionality can be found in its documentation at http://pyscestoolbox.readthedocs.io.
