# Supplementary material for: Delving deeper: Relating the behaviour of a metabolic system to the properties of its components using symbolic metabolic control analysis
Source: PLoS One. 2018 Nov 28;13(11):e0207983. doi: 10.1371/journal.pone.0207983 (PMC6261606; doi:10.1371/journal.pone.0207983)
Supplement: S1 Fig — This figure is a recreation of Fig 6 in the main text that shows the effects of replacing reaction 11 with a hypothetical reaction with a 1000-fold larger Keq value. For more details see the original figure. (A) The elasticity and flux factors that constitute the multipliers T4 and T6. All components of these two multipliers besides the shared J3εϕCv6εAcalv7 factor and εAcetv10 belonging to T4, decreased in magnitude due to the increase Keq. (B) The elasticity coefficient εAcetv11 split into its binding and mass action components. The mass action component was altered such that it had a value of ≈ 1 for the most of the tested range of ϕN values. The insert shows εAcetv11 on an expanded scale. (PDF) [file pone.0207983.s002.pdf]

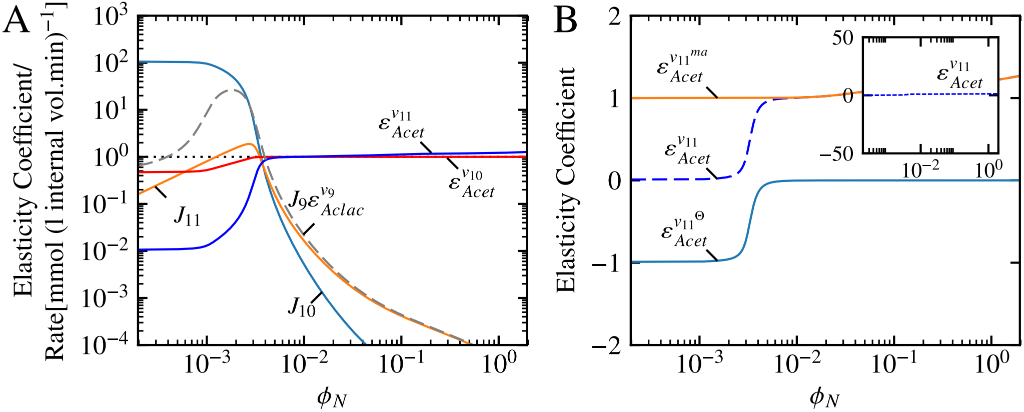

S1 Fig.: The flux and elasticity components of T4 and T6 as functions of ϕ_N after alteration of K_{eq} of reaction 11. This figure is a recreation of Fig. 6 in the main text that shows the effects of replacing reaction 11 with a hypothetical reaction with a 1000-fold larger K_{eq} value. For more details see the original figure. (A) The elasticity and flux factors that constitute the multipliers T4 and T6. All components of these two multipliers besides the shared $J_3\varepsilon^{v_6}_{\phi_C}\varepsilon^{v_7}_{Acal}$ factor and $\varepsilon^{v_{10}}_{Acet}$ belonging to T4, decreased in magnitude due to the increase K_{eq}. (B) The elasticity coefficient $\varepsilon^{v_{11}}_{Acet}$ split into its binding and mass action components. The mass action component was altered such that it had a value of ≈ 1 for the most of the tested range of ϕ_N values. The insert shows $\varepsilon^{v_{11}}_{Acet}$ on an expanded scale.
