# Supplementary material for: Delving deeper: Relating the behaviour of a metabolic system to the properties of its components using symbolic metabolic control analysis
Source: PLoS One. 2018 Nov 28;13(11):e0207983. doi: 10.1371/journal.pone.0207983 (PMC6261606; doi:10.1371/journal.pone.0207983)
Supplement: S2 Fig — This figure is a recreation of Fig 3 in the main text that shows the effects of replacing reaction 11 with a hypothetical reaction with a 1000-fold larger Keq value. For more details see the original figure. (A) The most important control patterns shown in relation to the value of Cv3J6 and the value of their total sum. (B) The absolute percentage contribution of the most important control patterns relative to the absolute sum of the values of all Cv3J6 control patterns. Control patterns and Cv3J6 are indicated in the key. Comparing this figure to Fig 3 in the main text reveals them to be identical in spite of the change in reaction 11. (PDF) [file pone.0207983.s003.pdf]

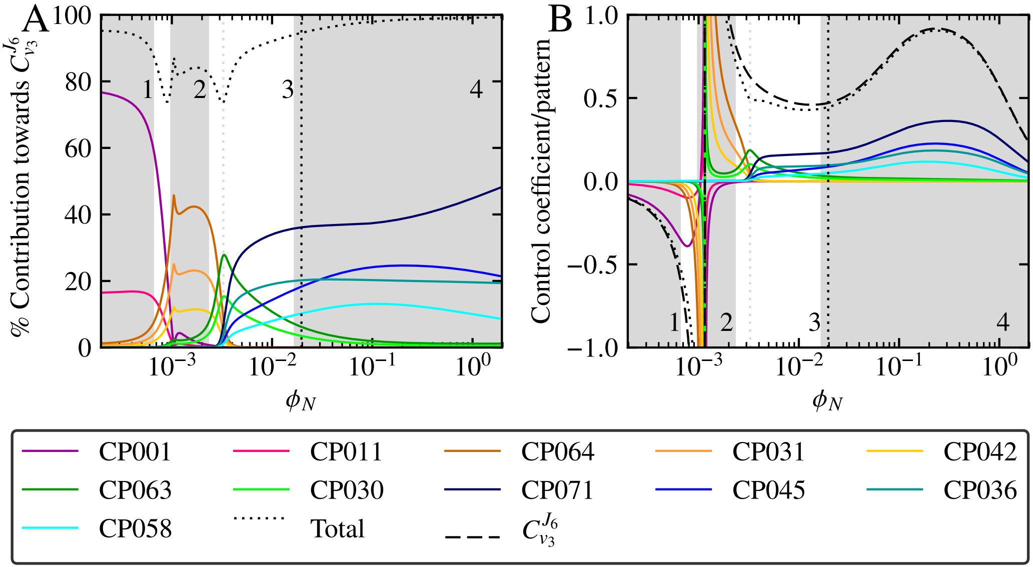

S2 Fig.: The most important control patterns of $C^{J_6}_{v_3}$ as functions of ϕ_N after alteration of K_{eq} of reaction 11. This figure is a recreation of Fig. 3 in the main text that shows the effects of replacing reaction 11 with a hypothetical reaction with a 1000-fold larger K_{eq} value. For more details see the original figure. (A) The most important control patterns shown in relation to the value of $C^{J_6}_{v_3}$ and the value of their total sum. (B) The absolute percentage contribution of the most important control patterns relative to the absolute sum of the values of all $C^{J_6}_{v_3}$ control patterns. Control patterns and $C^{J_6}_{v_3}$ are indicated in the key. Comparing this figure to Fig. 3 in the main text reveals them to be identical in spite of the change in reaction 11.
