# Supplementary material for: Delving deeper: Relating the behaviour of a metabolic system to the properties of its components using symbolic metabolic control analysis
Source: PLoS One. 2018 Nov 28;13(11):e0207983. doi: 10.1371/journal.pone.0207983 (PMC6261606; doi:10.1371/journal.pone.0207983)
Supplement: S3 Fig — This figure is a recreation of Fig 4 in the main text that shows the effects of replacing reaction 11 with a hypothetical reaction with a 1000-fold larger Keq value. For more details see the original figure. (A) Values of the backbone patterns (A–F) scaled by the control coefficient common denominator (Σ) of this pathway. Comparing (A) to Fig 4A shows that C and A increased after the change. (B) Values of the multiplier patterns consisting of components from the bottom half of the reaction scheme in Fig 2 (B1–B6). These multiplier patterns were unaffected by the alteration of reaction 11. (C) Values of the multiplier patterns consisting of components from the top half of the reaction scheme (T1–T7). Comparing (C) to Fig 4C shows that all T multipliers decreased after the increase in Keq (D) CP063 and CP071 together with their constituent scaled backbone and multiplier patterns. (E) CP001 and CP071. (F) The constituent scaled backbone and multiplier components of CP001 and CP071. From (D–F) it is clear that while both backbone patterns A and C and the T multipliers changed due to the alteration of reaction 11, these changes perfectly counteracted each other, thus resulting in no net change in the control pattern values of CP001, CP063 and CP071 (as well the rest of the 11 most important control patterns). It is interesting to note that backbone pattern B, which showed no change after perturbation of reaction 11, consists of elements from the same branches as backbones A and C and as the T multipliers. (PDF) [file pone.0207983.s004.pdf]

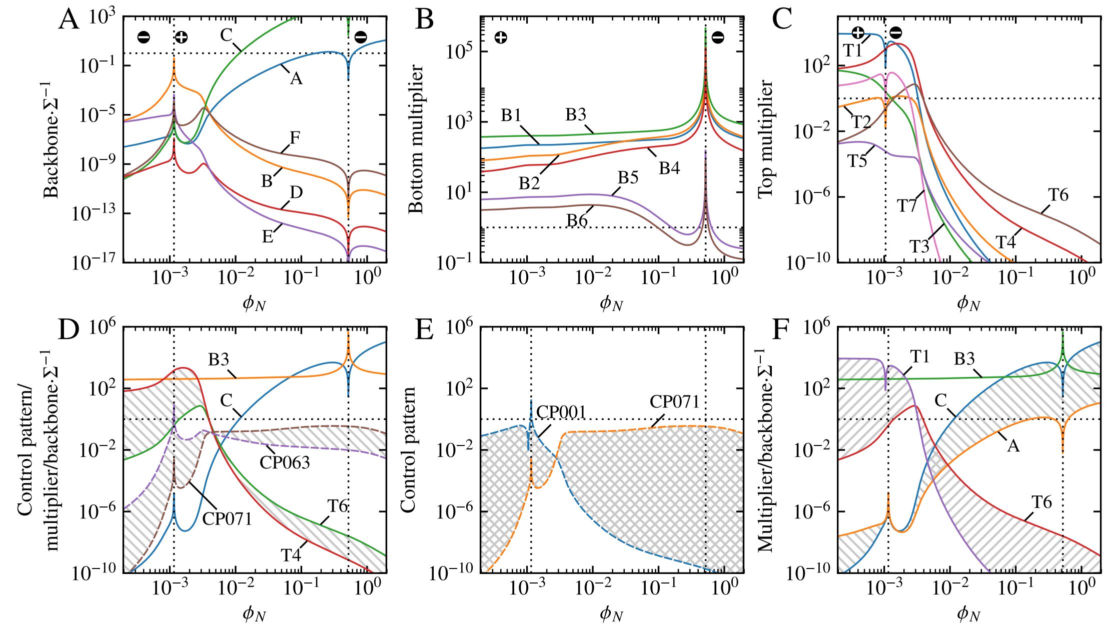

S3 Fig.: Backbone and multiplier patterns of the $C^{J_6}_{v_3}$ control patterns as functions of ϕ_N after alteration of K_{eq} of reaction 11.. This figure is a recreation of Fig. 4 in the main text that shows the effects of replacing reaction 11 with a hypothetical reaction with a 1000-fold larger K_{eq} value. For more details see the original figure. (A) Values of the backbone patterns (A–F) scaled by the control coefficient common denominator (Σ) of this pathway. Comparing (A) to Fig. 4A shows that C and A increased after the change. (B) Values of the multiplier patterns consisting of components from the bottom half of the reaction scheme in Fig. 2 (B1–B6). These multiplier patterns were unaffected by the alteration of reaction 11. (C) Values of the multiplier patterns consisting of components from the top half of the reaction scheme (T1–T7). Comparing (C) to Fig. 4C shows that all T multipliers decreased after the increase in K_{eq} (D) CP063 and CP071 together with their constituent scaled backbone and multiplier patterns. (E) CP001 and CP071. (F) The constituent scaled backbone and multiplier components of CP001 and CP071. From (D–F) it is clear that while both backbone patterns A and C *and* the T multipliers changed due to the alteration of reaction 11, these changes perfectly counteracted each other, thus resulting in no net change in the control pattern values of CP001, CP063 and CP071 (as well the rest of the 11 most important control patterns). It is interesting to note that backbone pattern B, which showed no change after perturbation of reaction 11, consists of elements from the same branches as backbones A and C and as the T multipliers.
