# Supplementary material for: Delving deeper: Relating the behaviour of a metabolic system to the properties of its components using symbolic metabolic control analysis
Source: PLoS One. 2018 Nov 28;13(11):e0207983. doi: 10.1371/journal.pone.0207983 (PMC6261606; doi:10.1371/journal.pone.0207983)
Supplement: S4 Fig — Instead of directly altering the value of ϕN, the activity of NADH oxidase was modulated to produce the results shown here. Control patterns were chosen according to the criteria described in the main text (which yielded percentages of 5% and 3% for the two cut-off criteria). (A) The most important control patterns shown in relation to the value of Cv3J6 and the value of their total sum. While the control coefficient and its constituent patterns follow a similar pattern as those of the fixed-NADH/NAD+ model, there are clear differences in Cv3J6 between the two models (see Fig 3 in the main text). (B) The absolute percentage contribution of the most important control patterns relative to the absolute sum of the values of all Cv3J6 control patterns. In spite of differences between Cv3J6 in the two models, the cumulative effect of the 20 most important control patterns in the free-NADH/NAD+ model closely mirrors that of the 11 most important control patterns in the fixed-NADH/NAD+ model. Control patterns and Cv3J6 are indicated in the key. While control patterns can be subdivided into similar dominant groups as in the fixed-NADH/NAD+ model, those groups (along with associated colour coding as shown in Fig 3 in the main text) are not indicated here. The switch from negative control coefficient values to positive values indicates indicates the reversal of direction of J6 flux. The black dotted vertical line indicates the steady-state value of ϕN in the reference model. (PDF) [file pone.0207983.s005.pdf]

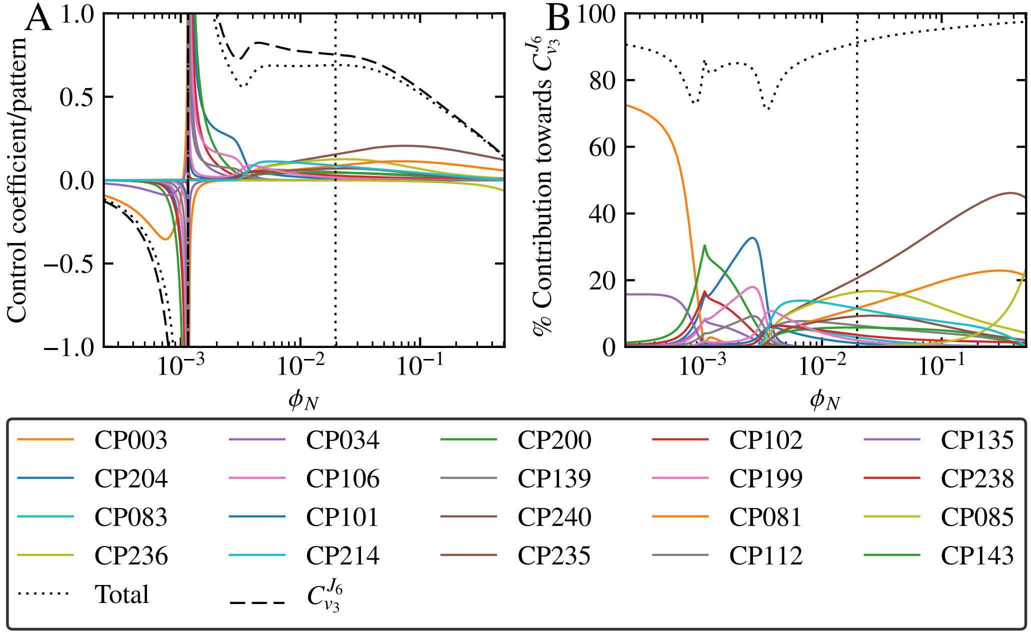

S4 Fig.: The most important control patterns of $C_{v_3}^{J_6}$ as functions of ϕ_N in the free-NADH/NAD$^+$ model. Instead of directly altering the value of ϕ_N, the activity of NADH oxidase was modulated to produce the results shown here. Control patterns were chosen according to the criteria described in the main text (which yielded percentages of 5% and 3% for the two cut-off criteria). (A) The most important control patterns shown in relation to the value of $C_{v_3}^{J_6}$ and the value of their total sum. While the control coefficient and its constituent patterns follow a similar pattern as those of the fixed-NADH/NAD$^+$ model, there are clear differences in $C_{v_3}^{J_6}$ between the two models (see Fig. 3 in the main text). (B) The absolute percentage contribution of the most important control patterns relative to the absolute sum of the values of all $C_{v_3}^{J_6}$ control patterns. In spite of differences between $C_{v_3}^{J_6}$ in the two models, the cumulative effect of the 20 most important control patterns in the free-NADH/NAD$^+$ model closely mirrors that of the 11 most important control patterns in the fixed-NADH/NAD$^+$ model. Control patterns and $C_{v_3}^{J_6}$ are indicated in the key. While control patterns can be subdivided into similar dominant groups as in the fixed-NADH/NAD$^+$ model, those groups (along with associated colour coding as shown in Fig. 3 in the main text) are not indicated here. The switch from negative control coefficient values to positive values indicates indicates the reversal of direction of J_6 flux. The black dotted vertical line indicates the steady-state value of ϕ_N in the reference model.
