# Supplementary material for: Delving deeper: Relating the behaviour of a metabolic system to the properties of its components using symbolic metabolic control analysis
Source: PLoS One. 2018 Nov 28;13(11):e0207983. doi: 10.1371/journal.pone.0207983 (PMC6261606; doi:10.1371/journal.pone.0207983)
Supplement: S5 Fig — Each plot shows the same basic results, with each being generated in different manner. (A) These results were generated by modulating the activity of NADH oxidase in order to indirectly alter ϕN in the free-NADH/NAD+ model. (B) The results of (A) plotted against ϕN. (C) This rate characteristic was generated in the fixed-NADH/NAD+ model by directly modulating ϕN over the same range as produced by modulating Vmax13 in (A). Comparing (B) and (C) reveals that in spite the structural difference between the two models caused by fixing ϕN, there is no difference in the flux responses of the two models, besides for J13 which consists solely of NADH oxidase. There are, however, differences in the control coefficients as demonstrated in S4 Fig. (PDF) [file pone.0207983.s006.pdf]

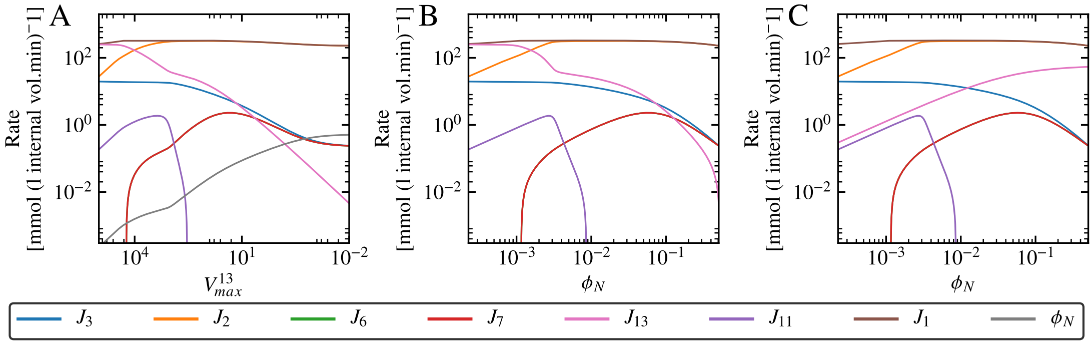

S5 Fig.: Rate characteristic plots of the reaction block fluxes of ϕ_N. Each plot shows the same basic results, with each being generated in different manner. (A) These results were generated by modulating the activity of NADH oxidase in order to indirectly alter ϕ_N in the free-NADH/NAD$^+$ model. (B) The results of (A) plotted against ϕ_N. (C) This rate characteristic was generated in the fixed-NADH/NAD$^+$ model by directly modulating ϕ_N over the same range as produced by modulating V_{max}^{13} in (A). Comparing (B) and (C) reveals that in spite the structural difference between the two models caused by fixing ϕ_N, there is no difference in the flux responses of the two models, besides for J_{13} which consists solely of NADH oxidase. There are, however, differences in the control coefficients as demonstrated in S4 Fig.
