# Supplementary material for: Delving deeper: Relating the behaviour of a metabolic system to the properties of its components using symbolic metabolic control analysis
Source: PLoS One. 2018 Nov 28;13(11):e0207983. doi: 10.1371/journal.pone.0207983 (PMC6261606; doi:10.1371/journal.pone.0207983)
Supplement: S1 Table — Each control pattern in the free-NADH/NAD+ model is, to some degree, equivalent to a control pattern in the fixed-NADH/NAD+ model and is expressed in terms of its counterpart (including additional factors) in the column “Expression”. Control patterns are arranged in the same groups and order as their equivalent control patterns in the fixed-NADH/NAD+ model according to their ranges of dominance as described in Table 2 of the main text. While each group for this model is equivalent to a group in the fixed-NADH/NAD+ model, here group 2 is subdivided into two smaller groups (2A and 2B) based on the difference of the control pattern shapes as shown in S4 Fig. The full control pattern numerator expressions are shown in the column “Full Expression”. (PDF) [file pone.0207983.s007.pdf]

S1 Table: Numerator expressions of the dominant control patterns of $C_{v_3}^{J_6}$ in the free-NADH/NAD$^+$ model. Each control pattern in the free-NADH/NAD$^+$ model is, to some degree, equivalent to a control pattern in the fixed-NADH/NAD$^+$ model and is expressed in terms of its counterpart (including additional factors) in the column "Expression". Control patterns are arranged in the same groups and order as their equivalent control patterns in the fixed-NADH/NAD$^+$ model according to their ranges of dominance as described in Table 2 of the main text. While each group for this model is equivalent to a group in the fixed-NADH/NAD$^+$ model, here group 2 is subdivided into two smaller groups (2A and 2B) based on the difference of the control pattern shapes as shown in S4 Fig. The full control pattern numerator expressions are shown in the column "Full Expression".

Group	Control Pattern	Expression	Full Expression
1	CP003	$\mathrm{CP001} \cdot \left(J_{13}\varepsilon^{v_{13}}_{\phi_N}\right)$	$J_1 J_{10} J_{12} J_{13} J_3 J_8 \varepsilon^{v_{10}}_{Acet}\varepsilon^{v_{12}}_{\phi_A}\varepsilon^{v_{13}}_{\phi_N}\varepsilon^{v_1}_{Pyr}\varepsilon^{v_5}_{Acp}\varepsilon^{v_6}_{\phi_N}\varepsilon^{v_7}_{Acal}\varepsilon^{v_8}_{Aclac}$
	CP034	$\mathrm{CP011} \cdot \left(J_{13}\varepsilon^{v_{13}}_{\phi_N}\right)$	$-J_1 J_{10} J_{12} J_{13} J_3 J_8 \varepsilon^{v_{10}}_{Acet}\varepsilon^{v_{12}}_{\phi_A}\varepsilon^{v_{13}}_{\phi_N}\varepsilon^{v_1}_{Pyr}\varepsilon^{v_4}_{Acp}\varepsilon^{v_6}_{\phi_N}\varepsilon^{v_7}_{Acal}\varepsilon^{v_8}_{Aclac}$
2A	CP200	$\mathrm{CP064} \cdot \left(J_{13}\varepsilon^{v_{13}}_{\phi_N}\right)$	$2J_{10} J_{12} J_{13} J_3 J_8 J_9 \varepsilon^{v_{10}}_{Acet}\varepsilon^{v_{12}}_{\phi_A}\varepsilon^{v_{13}}_{\phi_N}\varepsilon^{v_5}_{Acp}\varepsilon^{v_6}_{\phi_N}\varepsilon^{v_7}_{Acal}\varepsilon^{v_8}_{Pyr}\varepsilon^{v_9}_{Aclac}$
	CP102	$\mathrm{CP031} \cdot \left(J_{13}\varepsilon^{v_{13}}_{\phi_N}\right)$	$-2J_1 J_{10} J_{13} J_3 J_8 J_9 \varepsilon^{v_{10}}_{Acet}\varepsilon^{v_{13}}_{\phi_N}\varepsilon^{v_1}_{\phi_A}\varepsilon^{v_5}_{Acp}\varepsilon^{v_6}_{\phi_N}\varepsilon^{v_7}_{Acal}\varepsilon^{v_8}_{Pyr}\varepsilon^{v_9}_{Aclac}$
	CP135	$\mathrm{CP042} \cdot \left(J_{13}\varepsilon^{v_{13}}_{\phi_N}\right)$	$-2J_{10} J_{12} J_{13} J_3 J_8 J_9 \varepsilon^{v_{10}}_{Acet}\varepsilon^{v_{12}}_{\phi_A}\varepsilon^{v_{13}}_{\phi_N}\varepsilon^{v_4}_{Acp}\varepsilon^{v_6}_{\phi_N}\varepsilon^{v_7}_{Acal}\varepsilon^{v_8}_{Pyr}\varepsilon^{v_9}_{Aclac}$
2B	CP204	$\mathrm{CP064} \cdot \left(J_2\varepsilon^{v_2}_{\phi_N}\right)$	$2J_{10} J_{12} J_2 J_3 J_8 J_9 \varepsilon^{v_{10}}_{Acet}\varepsilon^{v_{12}}_{\phi_A}\varepsilon^{v_2}_{\phi_N}\varepsilon^{v_5}_{Acp}\varepsilon^{v_6}_{\phi_N}\varepsilon^{v_7}_{Acal}\varepsilon^{v_8}_{Pyr}\varepsilon^{v_9}_{Aclac}$
	CP106	$\mathrm{CP031} \cdot \left(J_2\varepsilon^{v_2}_{\phi_N}\right)$	$-2J_1 J_{10} J_2 J_3 J_8 J_9 \varepsilon^{v_{10}}_{Acet}\varepsilon^{v_1}_{\phi_A}\varepsilon^{v_2}_{\phi_N}\varepsilon^{v_5}_{Acp}\varepsilon^{v_6}_{\phi_N}\varepsilon^{v_7}_{Acal}\varepsilon^{v_8}_{Pyr}\varepsilon^{v_9}_{Aclac}$
	CP139	$\mathrm{CP042} \cdot \left(J_2\varepsilon^{v_2}_{\phi_N}\right)$	$-2J_{10} J_{12} J_2 J_3 J_8 J_9 \varepsilon^{v_{10}}_{Acet}\varepsilon^{v_{12}}_{\phi_A}\varepsilon^{v_2}_{\phi_N}\varepsilon^{v_4}_{Acp}\varepsilon^{v_6}_{\phi_N}\varepsilon^{v_7}_{Acal}\varepsilon^{v_8}_{Pyr}\varepsilon^{v_9}_{Aclac}$
3	CP199	$\mathrm{CP063} \cdot \left(J_{13}\varepsilon^{v_{13}}_{\phi_N}\right)$	$2J_{10} J_{12} J_{13} J_2 J_3 J_9 \varepsilon^{v_{10}}_{Acet}\varepsilon^{v_{12}}_{\phi_A}\varepsilon^{v_{13}}_{\phi_N}\varepsilon^{v_2}_{Pyr}\varepsilon^{v_5}_{Acp}\varepsilon^{v_6}_{\phi_N}\varepsilon^{v_7}_{Acal}\varepsilon^{v_9}_{Aclac}$
	CP238	$\mathrm{CP063} \cdot \left(2J_4\varepsilon^{v_4}_{\phi_C}\varepsilon^{v_6}_{\phi_N}/\varepsilon^{v_6}_{\phi_C}\right)$	$4J_{10} J_{12} J_2 J_3 J_4 J_9 \varepsilon^{v_{10}}_{Acet}\varepsilon^{v_{12}}_{\phi_A}\varepsilon^{v_2}_{Pyr}\varepsilon^{v_4}_{\phi_N}\varepsilon^{v_5}_{Acp}\varepsilon^{v_6}_{\phi_N}\varepsilon^{v_7}_{Acal}\varepsilon^{v_9}_{Aclac}$
	CP083	$\mathrm{CP063} \cdot \left(-2J_4\varepsilon^{v_4}_{\phi_C}\varepsilon^{v_6}_{Acal}\varepsilon^{v_7}_{\phi_N}/(\varepsilon^{v_6}_{\phi_C}\varepsilon^{v_7}_{Acal})\right)$	$-4J_{10} J_{12} J_2 J_3 J_4 J_9 \varepsilon^{v_{10}}_{Acet}\varepsilon^{v_{12}}_{\phi_A}\varepsilon^{v_2}_{Pyr}\varepsilon^{v_4}_{\phi_N}\varepsilon^{v_5}_{Acp}\varepsilon^{v_6}_{Acal}\varepsilon^{v_7}_{\phi_N}\varepsilon^{v_9}_{Aclac}$
	CP101	$\mathrm{CP030} \cdot \left(J_{13}\varepsilon^{v_{13}}_{\phi_N}\right)$	$-2J_1 J_{10} J_{13} J_2 J_3 J_9 \varepsilon^{v_{10}}_{Acet}\varepsilon^{v_{13}}_{\phi_N}\varepsilon^{v_1}_{\phi_A}\varepsilon^{v_2}_{Pyr}\varepsilon^{v_5}_{Acp}\varepsilon^{v_6}_{\phi_N}\varepsilon^{v_7}_{Acal}\varepsilon^{v_9}_{Aclac}$
4	CP240	$\mathrm{CP071} \cdot \left(2J_4\varepsilon^{v_4}_{\phi_C}\varepsilon^{v_6}_{\phi_N}/\varepsilon^{v_6}_{\phi_C}\right)$	$4J_{11} J_{12} J_2 J_3 J_4 J_9 \varepsilon^{v_{11}}_{Acet}\varepsilon^{v_{12}}_{\phi_A}\varepsilon^{v_2}_{Pyr}\varepsilon^{v_4}_{\phi_N}\varepsilon^{v_5}_{Acp}\varepsilon^{v_6}_{\phi_N}\varepsilon^{v_7}_{Acal}\varepsilon^{v_9}_{Aclac}$
	CP081	$\mathrm{CP036} \cdot \left(2J_4\varepsilon^{v_4}_{\phi_C}\varepsilon^{v_6}_{\phi_N}/\varepsilon^{v_6}_{\phi_C}\right)$	$-4J_1 J_{11} J_2 J_3 J_4 J_9 \varepsilon^{v_{11}}_{Acet}\varepsilon^{v_1}_{\phi_A}\varepsilon^{v_2}_{Pyr}\varepsilon^{v_4}_{\phi_N}\varepsilon^{v_5}_{Acp}\varepsilon^{v_6}_{\phi_N}\varepsilon^{v_7}_{Acal}\varepsilon^{v_9}_{Aclac}$
	CP085	$\mathrm{CP071} \cdot \left(-2J_4\varepsilon^{v_4}_{\phi_C}\varepsilon^{v_6}_{Acal}\varepsilon^{v_7}_{\phi_N}/(\varepsilon^{v_6}_{\phi_C}\varepsilon^{v_7}_{Acal})\right)$	$-4J_{11} J_{12} J_2 J_3 J_4 J_9 \varepsilon^{v_{11}}_{Acet}\varepsilon^{v_{12}}_{\phi_A}\varepsilon^{v_2}_{Pyr}\varepsilon^{v_4}_{\phi_N}\varepsilon^{v_5}_{Acp}\varepsilon^{v_6}_{Acal}\varepsilon^{v_7}_{\phi_N}\varepsilon^{v_9}_{Aclac}$
	CP236	$\mathrm{CP058} \cdot \left(2J_4\varepsilon^{v_1}_{\phi_N}\varepsilon^{v_5}_{\phi_A}/\varepsilon^{v_1}_{\phi_A}\right)$	$4J_1 J_{11} J_2 J_3 J_4 J_9 \varepsilon^{v_{11}}_{Acet}\varepsilon^{v_1}_{\phi_N}\varepsilon^{v_2}_{Pyr}\varepsilon^{v_4}_{Acp}\varepsilon^{v_5}_{\phi_A}\varepsilon^{v_6}_{\phi_N}\varepsilon^{v_7}_{Acal}\varepsilon^{v_9}_{Aclac}$
	CP214	$\mathrm{CP071} \cdot \left(J_{13}\varepsilon^{v_{13}}_{\phi_N}\right)$	$2J_{11} J_{12} J_{13} J_2 J_3 J_9 \varepsilon^{v_{11}}_{Acet}\varepsilon^{v_{12}}_{\phi_A}\varepsilon^{v_{13}}_{\phi_N}\varepsilon^{v_2}_{Pyr}\varepsilon^{v_5}_{Acp}\varepsilon^{v_6}_{\phi_N}\varepsilon^{v_7}_{Acal}\varepsilon^{v_9}_{Aclac}$
	CP235	$\mathrm{CP036} \cdot \left(-2J_4\varepsilon^{v_4}_{\phi_C}\varepsilon^{v_6}_{Acal}\varepsilon^{v_7}_{\phi_N}/(\varepsilon^{v_6}_{\phi_C}\varepsilon^{v_7}_{Acal})\right)$	$4J_1 J_{11} J_2 J_3 J_4 J_9 \varepsilon^{v_{11}}_{Acet}\varepsilon^{v_1}_{\phi_A}\varepsilon^{v_2}_{Pyr}\varepsilon^{v_4}_{\phi_N}\varepsilon^{v_5}_{Acp}\varepsilon^{v_6}_{Acal}\varepsilon^{v_7}_{\phi_N}\varepsilon^{v_9}_{Aclac}$
	CP112	$\mathrm{CP036} \cdot \left(J_{13}\varepsilon^{v_{13}}_{\phi_N}\right)$	$-2J_1 J_{11} J_{13} J_2 J_3 J_9 \varepsilon^{v_{11}}_{Acet}\varepsilon^{v_{13}}_{\phi_N}\varepsilon^{v_1}_{\phi_A}\varepsilon^{v_2}_{Pyr}\varepsilon^{v_5}_{Acp}\varepsilon^{v_6}_{\phi_N}\varepsilon^{v_7}_{Acal}\varepsilon^{v_9}_{Aclac}$
	CP143	$\mathrm{CP045} \cdot \left(J_{13}\varepsilon^{v_{13}}_{\phi_N}\right)$	$-2J_{11} J_{12} J_{13} J_2 J_3 J_9 \varepsilon^{v_{11}}_{Acet}\varepsilon^{v_{12}}_{\phi_A}\varepsilon^{v_{13}}_{\phi_N}\varepsilon^{v_2}_{Pyr}\varepsilon^{v_4}_{Acp}\varepsilon^{v_6}_{\phi_N}\varepsilon^{v_7}_{Acal}\varepsilon^{v_9}_{Aclac}$
